# *gyrA* ser83 mutation among fluoroquinolone-resistant *Salmonella enterica* serovars from enteric fever patients in tertiary care hospital, Kathmandu

**DOI:** 10.1186/s12866-022-02456-7

**Published:** 2022-02-10

**Authors:** Prashanna Adhikari, Roshani Maharjan, Subash Paudel, Bikram Malla, Pradeep Kumar Shah, Anup Bastola, Upendra Thapa Shrestha

**Affiliations:** 1 Department of Microbiology, Tri-Chandra Multiple Campus, Tribhuvan University, Ghantaghar, Kathmandu Nepal; 2 Central Department of Microbiology, Tribhuvan University, Kirtipur, Kathmandu Nepal; 3 Sukraraj Tropical and Infectious Disease Hospital, Teku, Kathmandu Nepal

**Keywords:** Enteric fever, *Salmonella enterica*, Fluoroquinolone-resistant, *gyrA*, ser83 mutation

## Abstract

**Background:**

The management of enteric fever through antibiotics is difficult these days due to the emerging resistance of *Salmonella* to various antimicrobial agents. The development of antimicrobial resistance is associated with multiple factors including mutations in the specific genes. To know the current status of mutation-mediated fluoroquinolone-resistance among *Salmonella enterica* serovars; Typhi, Paratyphi A, B and C, this study was focused on detecting *gyrA* ser83 mutation by restriction digestion analysis of *gyrA* gene using *HinfI* endonuclease.

**Results:**

A total of 948 blood samples were processed for isolation of *Salmonella* spp. and 3.4% of them were found to be positive for *Salmonella* growth. Out of the 32 *Salmonella* isolates, 2.2% were *S. *Typhi and 1.2% were *S. *Paratyphi A. More interestingly, we observed less than 5% of isolates were resistant to first-line drugs including chloramphenicol, cotrimoxazole and ampicillin. More than 80% of isolates were resistant to fluoroquinolones accounting for 84.4% to levofloxacin followed by 87.5% to ofloxacin and 100% to ciprofloxacin by disc diffusion methods. However, the minimum inhibitory concentration method using agar dilution showed only 50% of isolates were resistant to ciprofloxacin. A total of 3.1% of isolates were multidrug-resistant. Similarly, 90.6% of the *Salmonella* isolates showed *gyrA* ser83 mutation with resistance to nalidixic acid.

**Conclusions:**

The increased resistance to fluoroquinolones and nalidixic acid in *Salmonella* isolates in our study suggests the use of alternative drugs as empirical treatment. Rather, the treatment should focus on prescribing first-line antibiotics since we observed less than 5% of *Salmonella* isolates were resistant to these drugs.

## Background

Enteric fever is an acute, life-threatening febrile infection caused by *Salmonella enterica* serovars; Typhi, Paratyphi A, B and C. Symptoms may vary from mild to severe and usually begin 6 to 30 days after exposure [1]. These isolates are highly adapted infections to the human population, having no animal and environmental reservoirs. Enteric fever was once considered a major cause of morbidity and mortality throughout the world accounting for approximately 15% case fatality rate. World Health Organization (WHO) estimates the annual global incidence of enteric fever is between 11 and 21 million cases and approximately 128,000 to 161,000 deaths [2]. In addition, the infection is more prevalent in developing countries with poor sanitation including Nepal. As per the annual report published by the Department of Health Service (DoHS), Ministry of Health and Population, Nepal, enteric fever was still one of the top ten causes for inpatient morbidity (8.48%) [3]. Nepal remains endemic to enteric fever for many years. *S. *Typhi and *S. *Paratyphi A are the two most predominant bacterial pathogens in the blood cultures of enteric febrile patients in Nepal.

Although enteric fever is endemic in many developing countries including Nepal, effective antimicrobial therapy has significantly reduced morbidity and mortality among the infected cases. *S. *Typhi and *S. *Paratyphi A, B and C are susceptible to a variety of antibiotics in vitro testing. However, in vivo responses are not always similar to in vitro susceptibility. It is very difficult to predict accurately because of their predominantly intracellular location within phagocytic cells. These isolates were usually sensitive to the fluoroquinolone antibiotics which were rapidly bactericidal than the third-generation cephalosporins. Unfortunately, the resistance to fluoroquinolones has now developed in *S. *Typhi and *S. *Paratyphi isolates from central Asia, southern India and Vietnam [4–7]. The emerging resistance to fluoroquinolones adds another burden to manage enteric febrile cases. This is because of extensive use of fluoroquinolones for the treatment of infections caused by multidrug-resistant *Salmonella* spp. [8]. *S. enterica* has developed resistance to fluoroquinolones in many different ways including inactivation of drug, reduced membrane permeability, alteration of the target site and active efflux [9]. Likewise, single point mutations in the “quinolone resistance determining region” of the *gyrA* gene are usually associated with a mechanism of fluoroquinolone resistance [9]. The mutation may occur in *gyrA* when there is a change in the amino acid sequence either by addition or deletion leading to the development of resistance. The most commonly identified mutation has been a serine to phenylalanine substitution at position 83. Less common mutations have been reported as aspartate to tyrosine or glycine at position 87 [10, 11]. Moreover, the reduced susceptibility to the fluoroquinolones has also been reported in *Salmonella* isolates without any mutations in *gryA* gene suggesting an alternative way of resistance. The mutation in *gryA* gene is not only responsible for the reduced susceptibility to the fluoroquinolones but also increases the minimum inhibitory concentration (MIC) breakpoint values to fluoroquinolone antibiotics [12]. Hence, it is necessary to evaluate MIC break points to those antibiotics regularly. The susceptibility testing of *Salmonella* isolates to a variety of antibiotics are equally important to know the current antimicrobial-resistant status and prescribing potential drugs for disease management. This study was therefore conducted to determine the antibiogram patterns of *S. *Typhi and *S. *Paratyphi isolated in blood cultures of enteric febrile patients. Our study also estimated the fluoroquinolones resistance due to mutation of *gryA* gene at ser83 position using restriction digestion. Finally, we have also recommended some potential antibiotics to manage fluoroquinolones resistant *Salmonella* infections.

## Methods

### Study design, duration, and site

A hospital-based cross-sectional study was conducted from February to July 2019 among the clinically suspected patients with enteric fever visiting Sukraraj Tropical and Infectious Disease Hospital, Kathmandu, Nepal.

### Inclusion and exclusion criteria

The patients suspected of enteric fever from all sexes of greater than five years who gave written consent to participate in the study were included for data and sample collection. The collected samples with visible signs of contamination or insufficient volume were excluded.

### Consent from the patients

Written consent was taken from each participant. The patients’ information including demographic characteristics, travel history and clinical history were collected using a structured questionnaire.

### Sample collection

About 8–10 ml venous blood sample was collected from each adult patient and 3–5 ml from each child patient [13, 14]. A total of 948 blood samples from the patients were processed during the study period. 5 ml of blood from each adult patient and 3 ml from each child patient were inoculated in a BACTEC culture bottle and incubated at 37 °C for up to five days. The remaining blood samples were used for routine laboratory diagnosis including hematology, serology and biochemical investigation. The culture bottles showing bacterial growth were sub-cultured on Mac-Conkey agar (MA), Xylose lysine deoxycholate agar (XLD) and Blood agar (BA). The isolated colonies were further identified as per standard microbiological techniques including colony morphology, staining reaction, biochemical characteristics and serotyping method [15, 16].

### Antimicrobial susceptibility testing

Antimicrobial susceptibility testing of *Salmonella* isolates was performed by using the modified Kirby-Bauer disk diffusion method as recommended by CLSI guidelines. The antibiotics used in this study were ampicillin (AMP, 10 μg), azithromycin (AZM, 15 μg), cefixime (CFM, 5 μg), cefotaxime (CTX, 30 μg), ceftriaxone (CTR, 30 μg), ciprofloxacin (CIP, 5 μg), chloramphenicol (C, 30 μg), cotrimoxazole (COT, 25 μg), levofloxacin (LEV, 5 μg), nalidixic acid (NA, 30 μg) and ofloxacin (OF, 5 μg) from HiMedia [17]. MDR *Salmonella* species were identified based on the resistance to the three first-line drugs [18].

### Minimum inhibitory concentration (MIC) of ciprofloxacin

*Salmonella enterica* isolates were further subjected to the minimum inhibitory concentration of ciprofloxacin by the agar dilution method using the antibiotic concentration of 1280 μg/ml. *Salmonella* ATCC 35664 was used for quality control. Muller Hinton Agar (MHA) plates were prepared with different concentrations of ciprofloxacin antibiotics (0.002 to 256 μg/ml) as per the recommendation in Andrews and CLSI guidelines [17, 19]. Standardized test inoculum was inoculated on antibiotic-MHA plates using a micropipette capable of delivering 2 μl volume with about 10^4^ cells per spot. Each test organism was inoculated in triplicate. QC strain was inoculated for the validity of the test for every batch of agar dilution. The plates incubated aerobically at 37 °C for 18 to 20 h, were observed for visible colonies to confirm the growth of the bacterium. Inoculation of the organism in an antibiotic-free plate was undertaken as a control [19].

### DNA extraction and PCR amplification of *gyrA* gene

Genomic DNA was extracted from *Salmonella* isolates by phenol:chloroform method and the presence of DNA was confirmed on 0.8% agarose gel electrophoresis at 120 V for one hour with 0.1 μg/ml ethidium bromide (EtBr) concentration [20]. The PCR amplification of *gyrA* gene was carried out using a set of specific primers; *gyrA*-F (5′- CGA GAG AAA TTA CAC CGG TCA-3′) and *gyrA*-R (5′- AGC CCT TCA ATG CTG ATG TC-3′) from Macrogen Inc., Korea [21]. A total of 25 μl PCR reaction mixture volume containing 21 μl of 1X PCR master mix (Qiagen), 3 μl of template DNA, 0.5 μl of *gyrA* forward primer and 0.5 μl of *gyrA* reverse primer was used for PCR amplification. DNA from *Salmonella* ATCC 35664 as positive control and no template as negative control were run simultaneously in each PCR cycle. The *gyrA* gene amplification was carried out at an initial denaturation of 95 °C for 15 min followed by 30 cycles of denaturation at 94 °C for 60 s, annealing at 57 °C for 90 s, extension at 72 °C for 60 s and a final extension at 72 °C for 7 min [21]. The amplified *gyrA* gene was confirmed by its molecular size of 610 bp on 1.5% agarose gel electrophoresis with 0.1 μg/ml EtBr concentration.

### Digestion of amplified PCR product of *gyrA* by *HinfI*

The amplified PCR product of *gyrA* gene was further digested by using restriction endonuclease; Fast *HinfI* from Thermofisher Scientific Inc. A total restriction digestion reaction of 30 μl containing 17 μl of nuclease-free water, 2 μl of fast digest green buffer, 10 μl of amplified *gyrA* product and 1 μl of fast digest *HinfI* enzyme was prepared in a tube and mixed properly. The tube was incubated at 37 °C in a water bath for five minutes and then placed into another water bath at 65 °C for 20 min for enzyme inactivation following the manufacture’s guidelines. The digested product was run through 2% agarose gel electrophoresis at 120 V for 60 min and visualized under a UV transilluminator. The digested products into two fragments of molecular sizes; 343 bp and 149 bp were considered as the mutation in *gyrA* gene while the digested products from non-mutated isolates were revealed into three fragments of 244 bp, 149 bp and 118 bp sizes. The mutated and non-mutated isolates were further compared with nalidixic acid-resistant (NAS) and nalidixic acid-sensitive (NAS) groups.

### Data analysis

The preliminary data was managed by MS Excel and later transcribed to Statistical Package for Social Science (SPSS) software (version25) for statistical analysis. A scatterplot diagram to determine the breakpoint of ciprofloxacin MIC was generated using WHONET 5.0.

## Results

### Total blood cultures and growth results

Out of 948 blood cultures, 76 (8.0%) samples were culture positive, in which collectively 32 (3.4%) samples were found to be positive for *Salmonella* spp. including *Salmonella *Typhi (*n* = 21) and *Salmonella *Paratyphi A (*n* = 11). For the remaining samples, 17 (1.8%) samples were positive for *Staphylococcus aureus*, 20 (2.1%) for Coagulase Negative Staphylococci (CONS) and 7 (0.7%) were positive for *Escherichia coli*. Since our study was focused on enteric febrile cases by *Salmonella* isolates, the other isolates were not further processed.

### Distribution of enteric fever cases based on the age of the patients

The highest rate of *Salmonella* was isolated from blood specimens of the age groups 5–20 years (1.6%; 15/945) and 21–35 years (1.6%; 15/945) followed by 36–50 age group (0.2%; 2/945 (*p* <  0.0001) (Table 1). No *Salmonella* infection was observed among patients of higher than 50 years. Similarly, the highest number of *S. * Typhi was isolated from male patients of age 5–20 years (9/32) followed by 21–35 years (6/32). On the other hand, the highest number of *S. *Paratyphi A was observed among male patients of age 21–35 (5/32) years followed by female patients of 5–20 years (2/32) and 21–35 years (2/32) (Table 2).

**Table 1:** Distribution of blood cultures and number of *S. enterica* isolates based on the age and sex group of patients

Age group (years)	Number of cases (%)			Number of ***S. enterica*** isolates (%)			***p***-value
	Male	Female	Total	Male	Female	Total	
5–20	86 (9.1)	59 (6.2)	145 (15.3)	10 (1.0)	5 (0.5)	15 (1.6)	< 0.0001
21–35	236 (24.9)	136 (14.3)	372 (39.2)	11 (1.2)	4 (0.4)	15 (1.6)	
36–50	130 (13.7)	120 (12.7)	250 (26.4)	1 (0.1)	1 (0.1)	2 (0.2)	
51–65	79 (8.3)	50 (5.3)	129 (13.6)	0	0	0	
66–80	25 (2.7)	21 (2.2)	46 (4.9)	0	0	0	
81–95	5 (0.5)	1 (0.1)	6 (0.6)	0	0	0	
**Total**	**561 (59.2)**	**387 (40.8)**	**948 (100)**	**22 (2.3)**	**10 (1.1)**	**32 (3.4)**

**Table 2:** Distribution of *S. *Typhi and *S. *Paratyphi A isolates among different ages and sex groups of patients

Age group (years)	Number of ***S. Typhi***			Number of ***S. ***Paratyphi A			Total ***Salmonella*** isolates
	Male	Female	Total	Male	Female	Total	
5–20	9	3	12	1	2	3	15
21–35	6	2	8	5	2	7	15
36–50	0	1	1	1	0	1	2
51–65	0	0	0	0	0	0	0
66–80	0	0	0	0	0	0	0
81–95	0	0	0	0	0	0	0
**Total**	**15**	**6**	**21**	**7**	**4**	**11**	**32**

### Antimicrobial susceptibility pattern of *Salmonella* isolates

None of the isolates of *Salmonella* were resistant to cefixime followed by 3.1% of the isolates were resistant to the antibiotics; ampicillin, chloramphenicol, cotrimoxazole and ceftriaxone. In the case of fluoroquinolones, 100% of the isolates were resistant to ciprofloxacin while 84.4% of *Salmonella* isolates were resistant to levofloxacin followed by 90.6% resistant to nalidixic acid and 87.5% non-susceptible to ofloxacin (Table 3). Out of 32 *Salmonella* isolates, only one *S. *Typhi was found to be multidrug-resistant.

**Table 3:** Antimicrobial susceptibility pattern of *Salmonella* isolates

Generation of antibiotics	Category of antibiotics	Antibiotics	Antibiotic Susceptibility Pattern (***n*** = 32)		
			Sensitive n (%)	Intermediate n (%)	Resistant n (%)
First	Fluoroquinolone	Nalidixic acid (30 μg)	3 (9.4)	0	29 (90.6)
Second	Macrolides	Azithromycin (15 μg)	30 (93.8)	2 (6.2)	0
	Fluoroquinolone	Ciprofloxacin (5 μg)	0 (0)	23 (71.9)	9 (28.1)
	Chloramphenicol (bacteriostatic)	Chloramphenicol (30 μg)	31 (96.9)	0	1 (3.1)
	Trimethoprim /sulfamethoxazole	Cotrimoxazole (25 μg)	31 (96.9)	0	1 (3.1)
	Fluoroquinolone	Ofloxacin (5 μg)	4 (12.5)	26 (81.2)	2 (6.3)
Third	Aminopenicillins	Ampicillin (10 μg)	31 (96.9)	0	1 (3.1)
	Cephalosporin	Cefixime (5 μg)	32 (100)	0	0
	Cephalosporin	Cefotaxime (30 μg)	23 (71.9)	4 (12.5)	5 (15.6)
	Cephalosporin	Ceftriaxone (30 μg)	31 (96.9)	1 (3.1)	0
	Fluoroquinolone	Levofloxacin (5 μg)	5 (15.6)	25 (78.1)	2 (6.3)

### Minimum inhibitory concentration value of ciprofloxacin

**Fig. 1:**
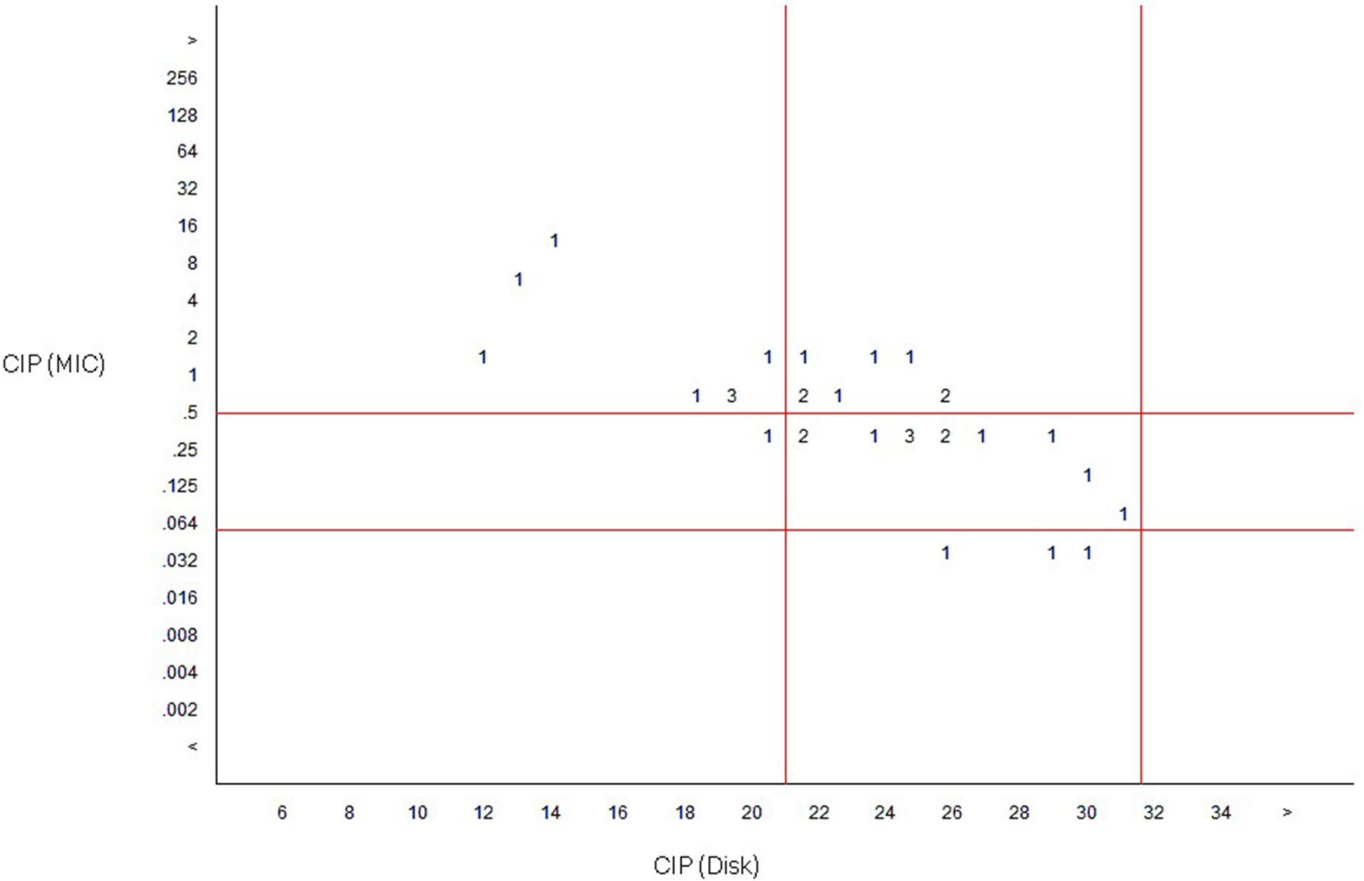
Scatterplot analysis of MIC values (μg/ml) vs disk diffusion values (Zone of Inhibition in mm) of ciprofloxacin (output from WHONET after analysis). Disk diffusion interpretative range; susceptible: ≥31 mm, intermediate: 21–30 mm, resistant: ≤20 mm. MIC interpretative range; susceptible: ≤0.06 μg/ml, intermediate: 0.12–0.5 μg/ml, resistant: ≥1 μg/ml. The numeric values; 1, 2 and 3 represent the number of *Salmonella* isolates resistant to the specific concentration of ciprofloxacin

The scatter plot correlating the MIC values and disk diffusion method of ciprofloxacin suggests the reduced susceptibility of *Salmonella* isolates. Out of nine resistant isolates by disk diffusion (zone of inhibition < 21 mm), eight were resistant to ciprofloxacin by MIC (MIC value ≥1 μg/ml) method and one showed reduced susceptibility (0.12–0.5 μg/ml). Similarly, out of 23 isolates that were intermediately resistant to ciprofloxacin by disk diffusion method, only eight isolates were found to be resistant by MIC, 12 isolates with reduced susceptibility and three were sensitive. A large number of isolates were found in the area of ciprofloxacin intermediate with slightly increased MIC values (Fig. 1).

### PCR amplification of *gyrA* and *HinfI* restriction digestion analysis of *gyrA*

**Fig. 2:**
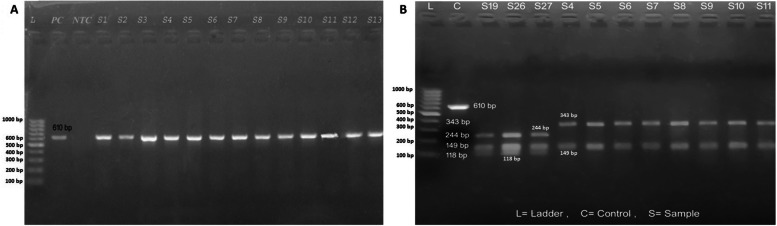
**A** Visualization of *gyrA* gene (610 bp) under UV transilluminator after electrophoresis (L: DNA ladder (GeneRuler 100 bp DNA Ladder, Thermo Fisher Scientific), PC: Positive control, NTC: No template control, S1-S13: Different *Salmonella* isolates); **B** Restriction pattern of *gyrA* after digestion by *HinfI* enzyme on UV transilluminator after electrophoresis. (Three fragments = non-mutated, two fragments = mutated) (The *HinfI* of *gyrA* gene in the original photo was not much clear mainly for smaller bands of 118 bp, 149 bp and 244 bp, hence, slight modifications including cropping of the image, labeling and increasing the contrast of bands have been done. The original photo can be submitted as supplementary on request)

*GyrA* gene of molecular weight of 610 bp was amplified in all 32 *Salmonella* isolates (Fig. 2A). Out of 32 isolates, *gyrA* ser83 mutation was observed in 90.6% [n=^22^] isolates showing two fragments of size 343 bp and 149 bp by *HinfI* digestion. 28.1% of mutated isolates were resistant and 62.5% showed reduced susceptibility to ciprofloxacin. All those isolates were resistant to nalidixic acid. In contrast, *gyrA* ser83 mutation was not detected in 9.4% [n=^3^] of *Salmonella* isolates. All three were sensitive to nalidixic acid antibiotic (Table 4, Fig. 2B).

**Table 4:** Distribution of *gyrA* restriction pattern based on the fluoroquinolone resistance pattern (n = 32)

Fluoroquinolone resistance phenotypes	NAR/NAS	No. of ***Salmonella*** spp (%)	MIC value for Ciprofloxacin (μg/ml)	***gyrA*** ser83 mutation
CIP_R_	NAR	9 (28.1)	0.5–16	Mutated (+)
CIP_I_	NAR	20 (62.5)	0.12–2	Mutated (+)
CIP_I_	NAS	3 (9.4)	0.06	Non-mutated (−)

**Note** CIP_R_ = Ciprofloxacin resistant**,** CIP_I_ = Ciprofloxacin intermediate, NAR = Nalidixic Acid Resistant, NAS = Nalidixic Acid Sensitive

## Discussion

Our study revealed 3.4% culture-positive blood samples to *Salmonella enterica*. This study agreed with a descriptive study done from 2011 to 2013 which reported 3.05% of culture-positive enteric febrile cases from different tertiary care hospitals in Kathmandu, Nepal [23]. However, another study by Adhikari et al. in 2012 reported a higher prevalence (7.6%) of enteric fever with *Salmonella* infections; *S. *Typhi and *S. *Paratyphi A from a tertiary care hospital in Kathmandu, Nepal [24]. The prevalence of enteric fever in Nepal seems to have fluctuated between 3 to 10% for the last decade without a significant increase or decrease in cases. Among age-wise distribution of enteric fever, the greatest number of cases were observed from the age group of 5–20 years and 21–35 years. Pokharel et al. had reported the highest number of enteric fever from the age group of 21–35 years in a hospital at Kathmandu [25].. Likewise, Bhetwal et al. also reported the highest number of *Salmonella* infections (*S. *Typhi and *S. *Paratyphi A) among the children of age 5–15 years in a community-based teaching hospital in Nepal in 2017 [13]. The higher rate of culture positivity among the age group of 5–35 years might be due to poor food hygiene and their dependency on out-sourcing foods [26]. Similarly, personal hygiene might be the reason for the higher infections among children.

We observed none of *Salmonella* isolates were resistant to cefixime and less than 5% resistance to the four antibiotics namely ampicillin, ceftriaxone, chloramphenicol and cotrimoxazole. The results also agree with the antibiogram report presented by Shrestha and Basnet in 2019 at Patan Hospital, Lalitpur, Nepal [27]. Although cefixime is a preferred drug of choice in developing countries due to the availability of an oral form for uncomplicated enteric fever [28], a study by Pandit et al. reported resistance to cefixime in vivo even if the isolates were susceptible in vitro study [29]. Although third-generation cephalosporins were found to be effective drugs for *Salmonella* isolates in our study, the increasing rate of extended-spectrum *β*-lactamase (ESBL) producing strains among other members of the *Enterobacteriaceae* family have added a huge challenge in empirical therapy [22, 30–32]. Those isolates could be the sources of ESBL genes to *Salmonella* isolates via horizontal transfer. Compared to a study by Maharjan et al. at the same hospital [33], we observed reduced susceptibility to fluoroquinolone antibiotics. The results were also supported by the data of Shrestha and Basnet [27]. The reason might be due to the extensive use of these drugs as therapeutic management of enteric fever. The rate of multidrug-resistant *Salmonella* was consistently low in the last two decades [22, 24, 34]. We also found only one MDR isolate while Maharajan et al. reported no MDR strain out of 40 *Salmonella* isolates in their study [33]. A low rate of MDR was supported by the findings of Britto et al. 2018 [35]. The abstain use of first-line antibiotics as empirical treatment for a longer period might have caused a loss of IncH1 plasmids leading to emerging back to sensitivity towards these drugs [36–38]. In addition, immunization could theoretically reduce the number of circulating MDR [35].

In our study, the scatterplot correlating the MIC and disk diffusion values of ciprofloxacin illustrates the reduced ciprofloxacin susceptibility among *Salmonella* isolates as compared to the studies at the same hospital [33, 39]. Compared to fluoroquinolone sensitive isolates, MIC values were commonly two or more dilutions higher (ciprofloxacin MIC 0.12 to 2 μg/ml for resistant strains compared with 0.5 to 16 μg/ml for intermediate resistant strains and 0.06 μg/ml for sensitive strains). When compared with nalidixic acid disc, the resistant strains showed ciprofloxacin MIC value of greater than 0.12 μg/ml. Nalidixic acid resistance hence serves as a surrogate marker for *gyrA* mutation associated with diminished fluoroquinolone susceptibility [40]. Out of 90.6% of NAR isolates, 40.6% of the *Salmonella* isolates had decreased susceptibility to ciprofloxacin with MIC value of 0.12 to 2 μg/ml and 50% isolates were resistant to ciprofloxacin with MIC value of up to 16 μg/ml. A similar study undertaken in India reported 47.5% resistance and 36.2% decreased susceptibility to ciprofloxacin among 97.5% of NAR *Salmonella* isolates, [41]. Nalidixic acid resistance showed a 100% predictive value for ciprofloxacin resistance as reported by Agrawal et al. [42]. Besides the *gyrA* gene mutation that occurred usually in the bacterial chromosome, the plasmid-mediated quinolone resistance mechanisms cannot be ignored [43].

Ser-83 mutation is the most commonly occurring point mutations among fluoroquinolone resistance strains. Over 90% of *gyrA* ser83 mutation was observed in *Salmonella* strains in our study which were similar to the studies by Khadka et al., 2021, Gopal et al. 2016 and Renuka et al. 2004 showing mutation rates of 95.7, 94 and 92.1% respectively [39, 44, 45]. Complete resistance or reduced susceptibility to fluoroquinolones is due to the mutation at ser-83 or asp-87 position of *gyrA* [35]. The mutations in the quinolone resistance determining regions of chromosomal genes such as *gyrA*, *gyrB*, *parC* and *parE* and plasmid-mediated *qnr, qepA* and *aacs* genes cause fluoroquinolones not to act and induce resistance in *Salmonella* spp. [43]. In our study, we observed that the strains with reduced susceptibility or complete resistance to fluoroquinolones retained ser-83 mutation. However, the isolates resistant to nalidixic acid and ciprofloxacin may have two or more mutations in the *gyrA, gyrB, parC, or parE* gene [46] which were not investigated in this study. In the case of patients with reduced susceptibility to fluoroquinolones for enteric fever, the infections can be managed with a higher dose of fluoroquinolones for a longer duration of time [47].

As a major study limitation, we only focused on *Salmonella* isolates and didn’t process for other bacterial pathogens isolated on the blood culture. Although there are many other mechanisms by which *Salmonella* may develop resistance to fluoroquinolones, due to limited time and budget, we couldn’t analyze all those mechanisms. We could only investigate *gyrA* ser83 mediated mutation among the isolates using restriction endonuclease *HinfI*. In addition, our study has shown the recent trend of the antimicrobial-resistant pattern of *Salmonella* isolates and suggested potential drugs for antimicrobial therapy.

## Conclusions

Since fluoroquinolones are the drugs of choice for the management of enteric fever, especially caused by *S. *Typhi and *S. *Paratyphi A in most developing countries, the decreased susceptibility to fluoroquinolones along with the accumulation of mutation in the ser83 position of *gyrA* gene may pose a threat to the disease management. In conclusion, our study suggests reintroducing ampicillin, chloramphenicol and cotrimoxazole drugs as empirical treatment for enteric fever as well as the use of third generation cephalosporins.

## Data Availability

The datasets used and/or analyzed during the current study will be available from the corresponding author on reasonable request at upendrats@gmail.com.

## Abbreviations

AST: Antibiotic Susceptibility testing
ATCC: American Type Culture Collection
BA: Blood Agar
CLSI: Clinical Laboratory Standard Institute
DOHS: Department Of Health Service
ESBL: Extended-Spectrum β-Lactamase
MA: Mac-Conkey Agar
MDR: Multidrug Resistance
MHA: Mueller Hinton Agar
MIC: Minimum Inhibitory Concentration
min: minute/s
NAR: Nalidixic acid-resistant
NAS: Nalidixic acid-sensitive
PCR: Polymerase chain reaction
QRDR: Quinolone Resistant Determining Region
RFLP: Restriction Fragment Length Polymorphism
SPSS: Statistical Package for Social Science
STIDHH: Sukraraj Tropical and Infectious Disease Hospital
WHO: World Health Organization
XLD: Xylose Lysine Deoxycholate Agar
